# Editorial: Omics technology in agriculture: molecular breeding for sustainable crop production

**DOI:** 10.3389/fpls.2023.1258701

**Published:** 2023-08-18

**Authors:** Zhiwen Chen, Corrinne E. Grover, Xiaoyang Ge, Xiaoxia Shangguan

**Affiliations:** ^1^ Engineering Research Center of Coal-based Ecological Carbon Sequestration Technology of The Ministry of Education, Key Laboratory of Graphene Forestry Application of National Forest and Grass Administration, Shanxi Datong University, Datong, China; ^2^ Department of Ecology, Evolution and Organismal Biology, Iowa State University, Ames, IA, United States; ^3^ Institute of Cotton Research, Chinese Academy of Agricultural Sciences, Anyang, China; ^4^ Institute of Cotton Research, Shanxi Agricultural University, Yuncheng, China

**Keywords:** crop production, omics-technologies, biotic or abiotic stresses, yield, quality

Omics technologies are high throughput technologies and belong to the concept of systems biology, which mainly includes genomics, transcriptomics, proteomics, metabolomics, phenomics, lipidomics, glycomics, and single-cell RNA transcriptome. Omics technologies bring numerous benefits that will provide new insights into basic research and crop breeding. It also has potential application to enable the discovery of new candidate genes to further verify their function for subsequent crop trait improvements. To advance research in the field of omics technology in agriculture, we are pleased to organize this Research Topic in Frontiers in Plant Science entitled “*Omics technology in agriculture: molecular breeding for sustainable crop production*.”

This Research Topic contains six papers that cover multiple perspectives of plant omics technology and functional gene verifications from rice, cotton, oil palm, pepper, and populus. Two papers focused on the genome-wide identification of important gene families, including R-SNARE genes in upland cotton and cytochrome P450 multigene family in pepper. Two other papers focused on the plant gene expression process and functional gene mining by using transcriptomics, metabolomics, and resequencing data with the genome-wide association method. In addition, two papers explored Na^+^ homeostasis under NaCl stress and plant non-coding RNAs’ function in pollen development and male sterility. According to their specific research directions, these six articles are summarized.

Omics technologies have developed rapidly and it has generated many datasets for different crops. Recent advances in cotton and pepper genomes have produced the resources necessary to analyze candidate gene families. For example, Li et al. performed a comprehensive analysis of the R-SNARE gene family in cotton and identified a key candidate gene of *GhVAMP72l* for resistance to drought stress. They totally identified 51 *Gh-R-SNARE* genes and the expression levels of nine genes were upregulated under drought stress conditions. Virus-induced gene silencing (VIGS) assay showed that *GhVAMP72l* might be the key candidate gene in response to drought stress in upland cotton (Li et al.). In addition, Hao et al. also performed a comprehensive identification of cytochrome P450 genes in pepper. In their study, they identified and classified 478 P450 genes from the pepper genome. Transcriptome data showed that P450 genes mainly play roles in placenta and pericarp development as well as in defensive and phytohormone response in pepper (Hao et al.).

Indeed, Zhang, S. et al. combined RNA-seq transcriptomics and LC-MS/MS metabolomics methods to show that four key enzyme genes SDR, FATA, FATB, and MFP in the rancidity of free fatty acids were significantly correlated with Palmitic acid, Stearic acid, Myristic acid, and Palmitoleic acid in oil palm fruit (Zhang, S. et al.). Further, Zhang, Q. et al. performed a GWAS study to show that *TUT1*, *Ghd7*, and *RGN1a* genes were in charge of the regulation of grain number per panicle in rice by re-analyzing the re-sequencing data. Among them, *RGN1a* was a novel gene to code a protein kinase in controlling grain number positively (Zhang, Q. et al.).

Wu et al. found that arbuscular mycorrhizal fungi (AMF) could promote growth and enhance the photosynthetic capacity and Na^+^ homeostasis in *Populus euphratica*. As salt stress increased Na^+^ contents and inhibited the growth of *P. euphratica*, the expression levels of *NHX1*, *HKT1*, and *SOS1* genes were upregulated to relieve the salt stress caused by Na^+^ homeostasis (Wu et al.). Finally, Nie et al. reviewed research progresses and molecular mechanisms of male sterility regulated by non-coding RNAs (ncRNAs) from the perspective of high-throughput sequencing technology. In their review, they summarized the critical ncRNAs related to the hormones that were involved in the processes of the stamen primordia differentiation, tapetum degradation, microspores formation, and pollen release. In addition, they also elaborated on the genetic mechanism of the miRNA-lncRNA-mRNA interaction network in regulating male sterility in plants (Nie et al.).

Omics technologies are conducive for researchers to study genome, transcriptome, proteome, metabolome, and phenome. Combining analysis of genetic and phenotypic data using omics approaches is an efficient way to identify genes responsible for important agronomic traits. Overall, the six papers in this Research Topic present the recent research advances in the fields of the plant genome, transcriptome, and metabolome based on omics technologies, providing important insights into plant biology. We thank the Frontiers in Plant Science for providing us with this Research Topic to display these results. We also thank all the contributors and reviewers for their contributions and valuable comments. We hope this Research Topic will be a useful resource for researchers in the field of studying plant functional genes based on omics technologies.

## Author contributions

ZC: Writing – original draft, Writing – review & editing. CG: Writing – original draft, Writing – review & editing. XG: Writing – original draft. XS: Writing – original draft.

